# Supplementary material for: Target-Site Mutations and Expression of ALS Gene Copies Vary According to Echinochloa Species
Source: Genes (Basel). 2021 Nov 22;12(11):1841. doi: 10.3390/genes12111841 (PMC8624184; doi:10.3390/genes12111841)
Supplement: Supplementary file 1 [file genes-12-01841-s001.zip › genes-1396714-supplementary.pdf]

Table S1. Some details on the populations included in the studies.

Species	Population code	Origin	Notes
ECH_CG	16S	Marzaro (PV)	Susceptible check
ECH_SS	44R	Bianzè (VC)	Treatments in the field: Beyond (imazamox); Facet (quinclorac); Viper (penoxsulam); Londax (bensulfuron)
ECH_SS	45R	San Vero Milis (OR)	Treatments in the field: Viper 2 (penoxsulam); Clincher (cyhalofop-butyl); Stam (propanil)
ECH_SS	46R	Ferrara (FE)	Treatments in the field: Viper 2 (penoxsulam)
ECH_CG	95R	Migliarino (FE)	Treated for several years with penoxsulam
ECH_CG	100R	Codigoro (FE)	Treatments in the field: Viper (penoxsulam); Clincher (cyhalofop-butyl); Aura (profoxydim)
ECH_ER	161S	Costanzana (VC)	Susceptible check

```
               CAPS-rbcL-F →
White (OR)  5'- CAACTGTTTGGACTGATGGACTTACCAGTCTTGATCGTTACAAAGGACGATGCTATCACATCGAGCCCGTT
Red (CG)    5'- CAACTGTTTGGACTGATGGACTTACCAGTCTTGATCGTTACAAAGGACGATGCTATCACATCGAGCCCGTT

White (OR)  CCTGGGGAGCCAGATCAATTTATCTGTTATATAGCTTATCCATTAGACCTATTTGAAGAGGGTTCTGTTACTAAC
Red (CG)    CCTGGGGAGCCAGATCAATATATCTGTTATATAGCTTATCCATTAGACCTATTTGAAGAGGGTTCTGTTACTAAC
                            TasI ↑
White (OR)  ATGTTTACTTCCATTGTGGGTAACGTATTTGGTTTCAAAGCCCTACGCGCTCTACGTTTGGAGGATCTACG – 3'
Red (CG)    ATGTTTACTTCCATTGTGGGTAACGTATTTGGTTTCAAAGCCCTACGCGCTCTACGTTTGGAGGATCTACG – 3'
                                                              ← CAPS-rbcL-R
```

Figure S1. Alignment of partial rbcL sequences of "white" (E. oryzicola, OR) and "red"(E. crus-galli, CG) species. The non-species-specific primers (CAPS-brcL-F and CAPS-rbcL-R) used to produce an amplicon of 217 bp are reported. The unique SNP detected in the sequence that creates a restriction site for the endonuclease TasI (AATT) in "white" samples is highlighted.

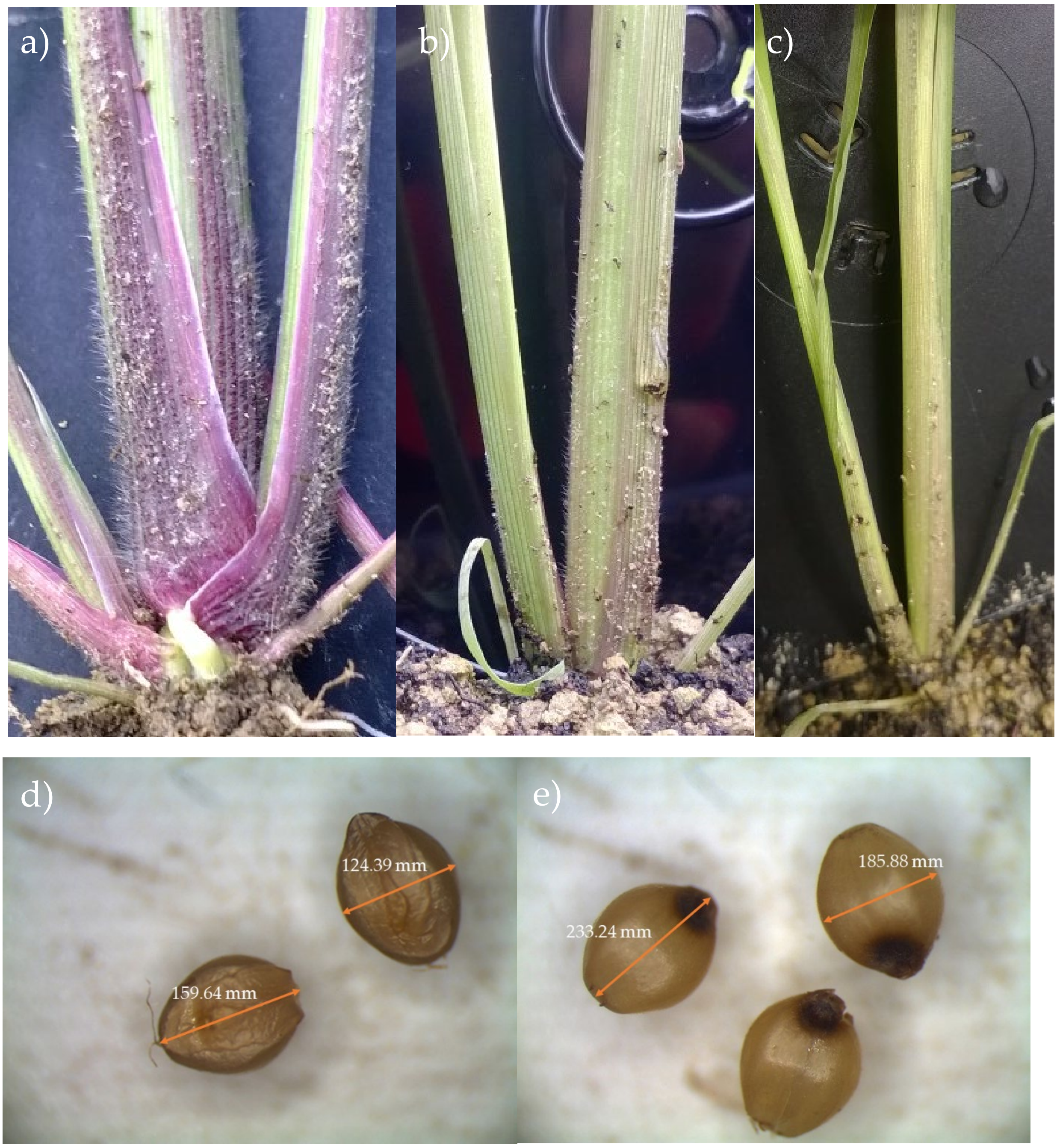

Figure S2. Morphological characteristics used to distinguish *Echinochloa* species. Stems' base of different *Echinochloa* plants: a) red base typical of *E. crus-galli* species, b) green base with hairs typical of "white" species *E. phyllopogon* and c) green hairless base typical of the "white" species *E. erecta*. Relative dimensions of the seeds: d) seeds of "red" species are typically smaller than e) seeds of the "white" species.

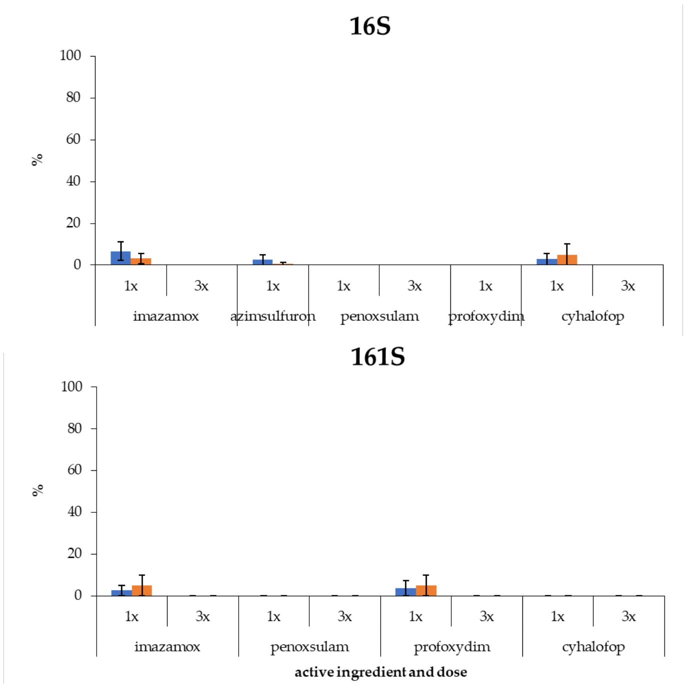

Figure S3. Screening test on susceptible populations. Plant survival (blue bars) and VEB (Visual Estimated Biomass) (orange bars) of the S *Echinochloa* spp. populations treated with herbicides having different sites of action: imazamox, azimsulfuron and penoxsulam are ALS inhibitors, whereas profoxydim and cyhalofop are ACCase inhibitors. The dose 1x corresponds to the recommended Italian field dose and 3x is 3 times that dose. The vertical thin bars represent the standard errors.

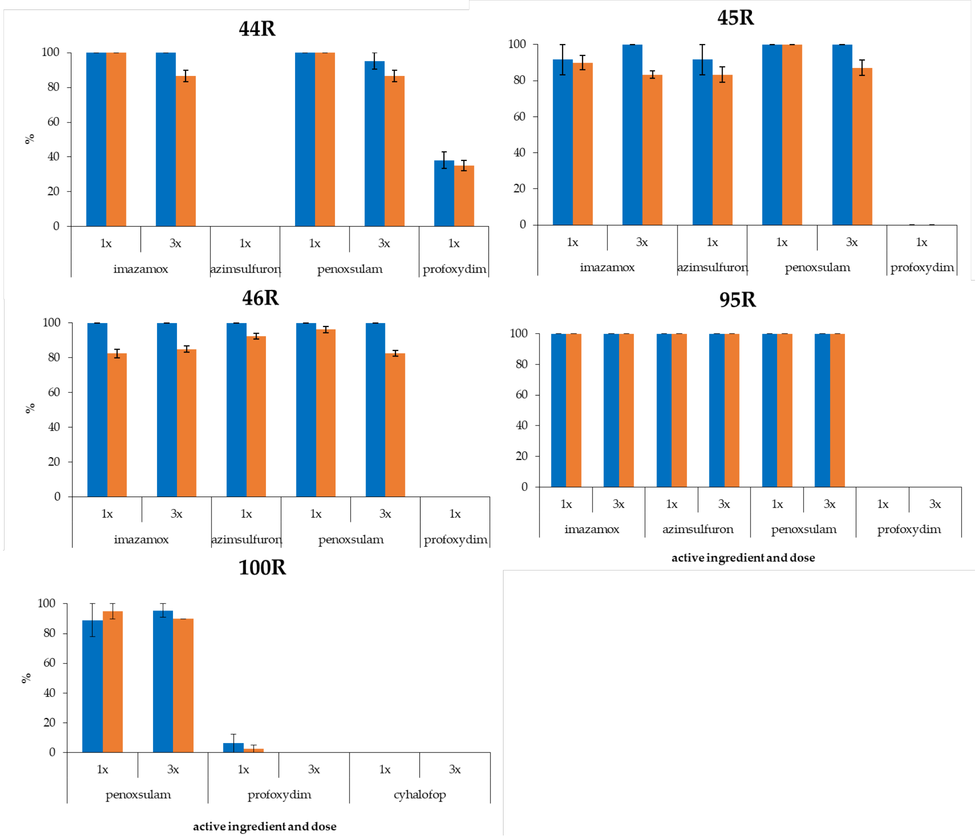

Figure S4. Screening test on resistant populations. Plant survival (blue bars) and VEB (Visual Estimated Biomass) (orange bars) of the R *Echinochloa* spp. populations treated with herbicides having different sites of action: imazamox, azimsulfuron

and penoxsulam are ALS inhibitors, whereas profoxydim and cyhalofop are ACCase inhibitors. The dose 1x corresponds to the recommended Italian field dose and 3x is three times that dose. The vertical thin bars represent the standard errors.

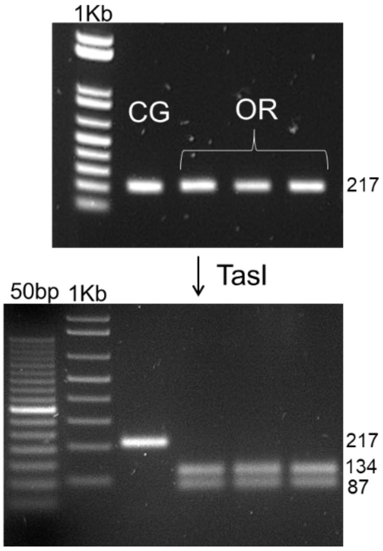

Figure S5. Scheme of gDNA-based molecular marker CAPS-rbcL. PCR on gDNA using primers CAPS-brcL-F and CAPS-rbcL-R produces amplicons of 217 bp. Digestion with TasI produces two bands in *E. oryzicola* (OR) samples, whereas it has no effect in *E. crus-galli* (CG) samples.

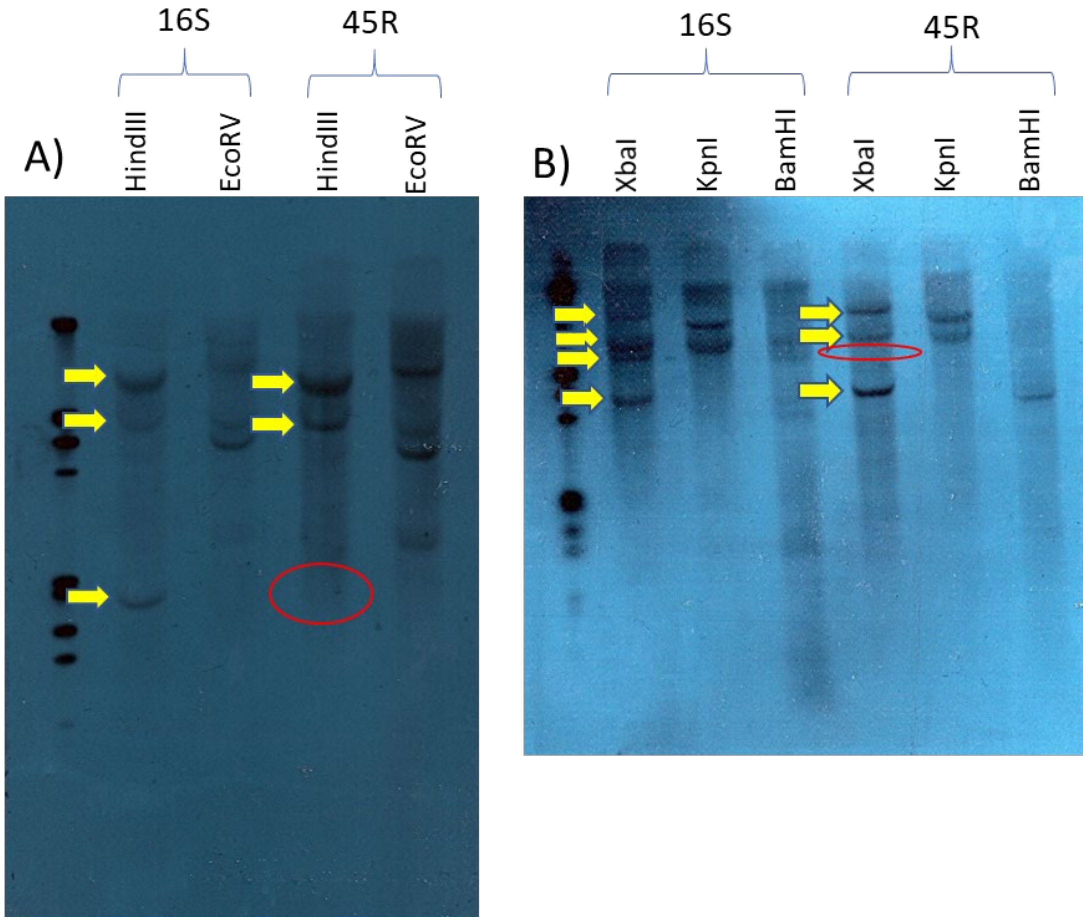

Figure S6. Results of the first two Southern blotting analyses. Southern blotting analysis on *ALS* gene of *E. crus-galli* (16S) and *E. oryzicola* (45R) genomic DNA extracted from leaf tissue. DNA samples of different populations were digested with A) the enzymes HindIII and EcoRV and B) XbaI, KpnI and BamHI. Arrows indicate the copies of the ALS gene in the different populations, circles indicate the missing gene copy in *E. oryzicola* population 45R.

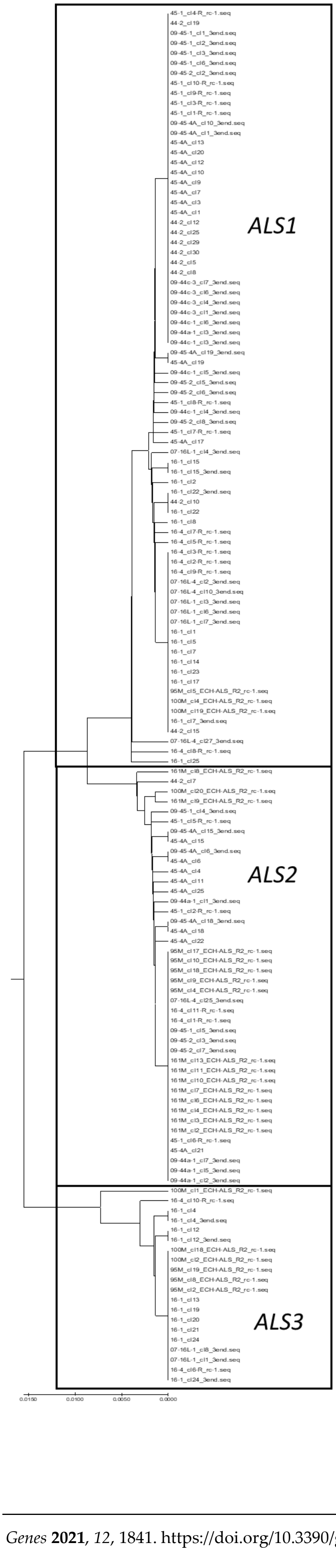
ALS1
ALS2
ALS3
0.0150
0.0100
0.0050
0.0000

Figure S7. Cladogram including sequences of both *E. crus-galli* and *E. oryzicola* populations. Cladogram obtained from UPMGA estimation of 138 *ALS* partial clone sequences starting from 3'-end using MEGA X®. *ALS1*, *ALS2* and *ALS3* are highlighted.

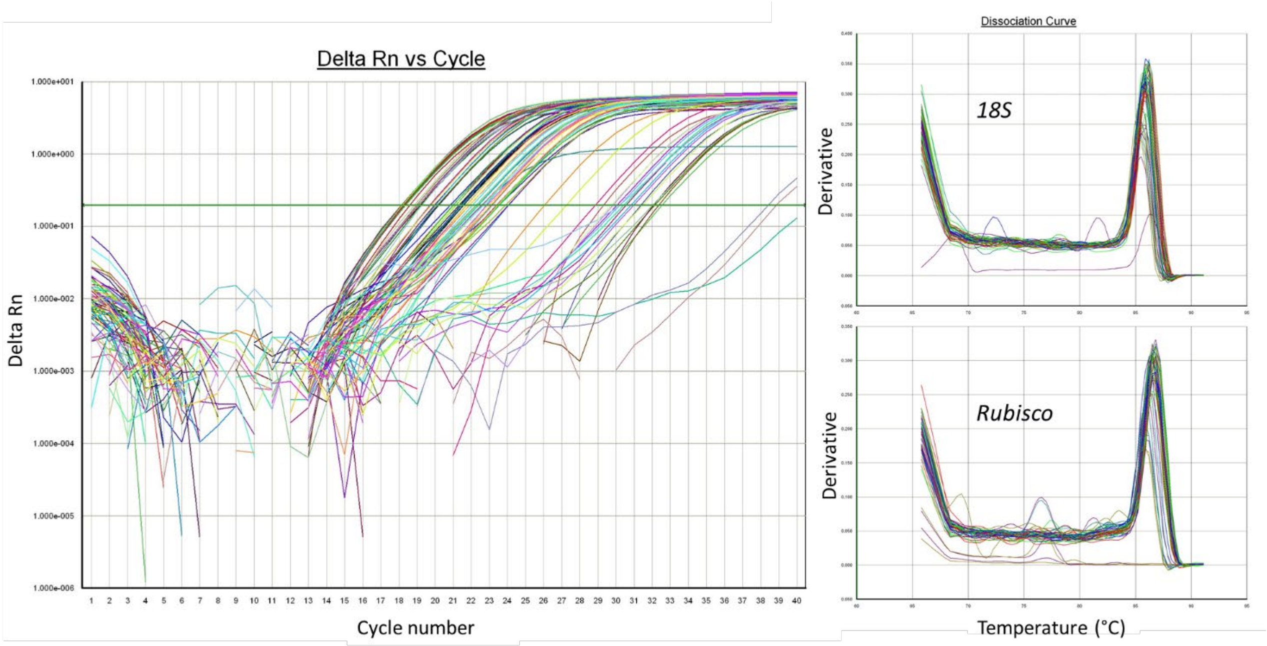

Figure S8. qPCR results for reference genes. Example of amplification plot (left) and dissociation curves (right) for the two reference genes selected for qPCR analyses, *18S* (top) and *Rubisco* (bottom). Plants of two populations of *Echinochloa* were included in this plate (16S and 45R) considering both herbicide-treated and not-treated plants (three plants per population and three replicates per plant). Three replicates of check (non-target-control, without cDNA) were also included.

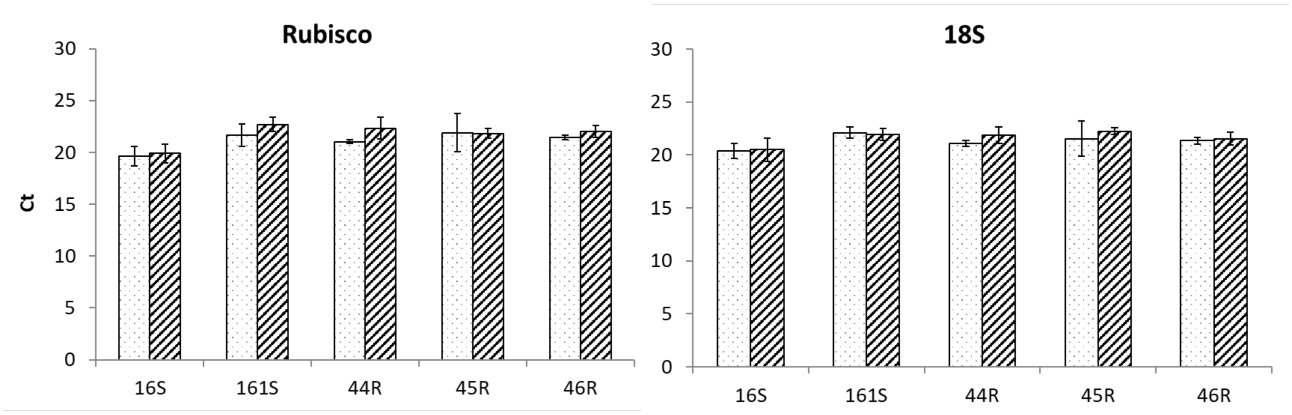

Figure S9. Ct of reference genes amplified on the different populations included in the experiment. Dotted bars represent the mean values of the treated plants, hatched bars represent the mean values of the untreated plants. Vertical thin bars represent the standard errors.

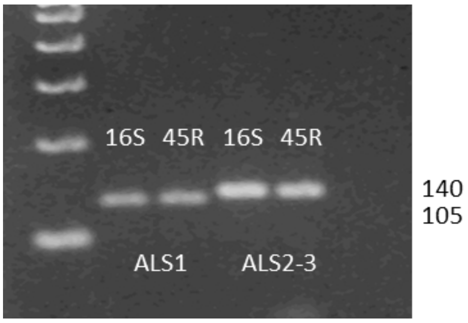

Figure S10. Digestion with the endonuclease FokI. PCR products were obtained using the allele specific primers F1_590Tm + R1_621 for the amplification of *ALS1* and F2_590Cm + R2_621 for the amplification of *ALS2-3* from the cDNA of 16S and 45R plants. The cutting site is present only in *ALS1*, yielding the 105 bp band visible only for the first two samples.

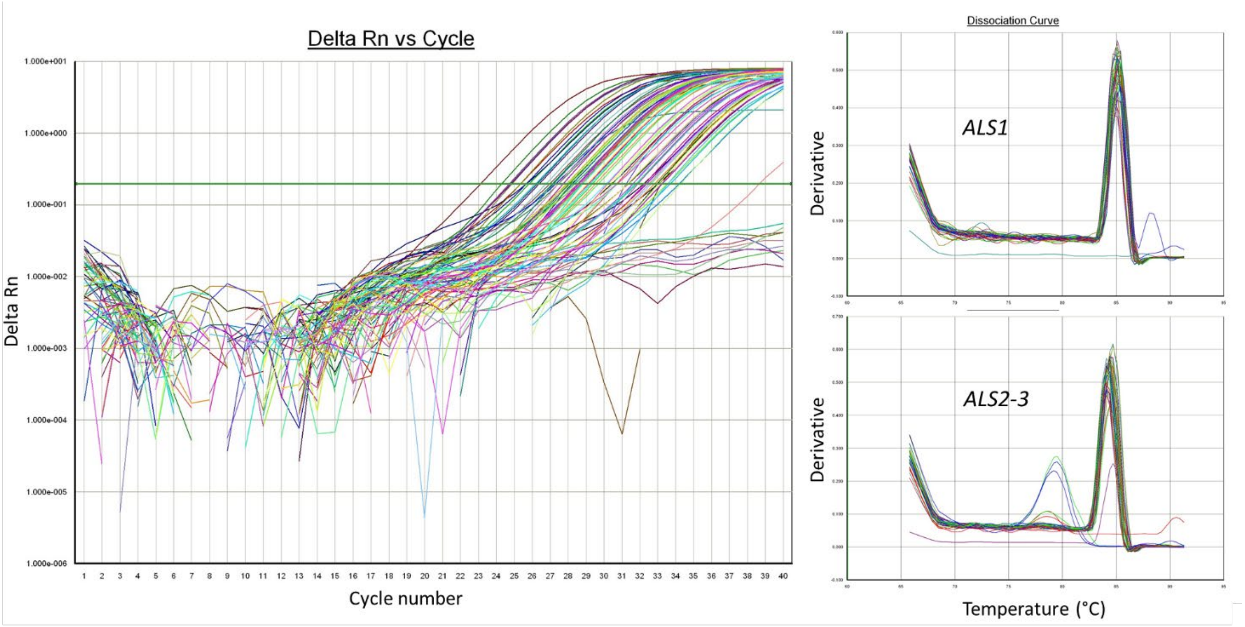

Figure S11. qPCR results for *ALS* genes. Example of amplification plot (left) and dissociation curves (right) for the ALS1 (top) and ALS2-3 (bottom) gene copies. Plants of two populations of *Echinochloa* were included in this plate (44R and 45R) considering both herbicide-treated and not-treated plants (three plants per population and three replicates for each plant). Three replicates of the check (non-target-control, without cDNA) were also included for each experiment.
